# The Effect of 6 Months’ Treatment With Pasireotide LAR on Glucose Metabolism in Patients With Resistant Acromegaly in Real-World Clinical Settings

**DOI:** 10.3389/fendo.2021.633944

**Published:** 2021-03-10

**Authors:** Przemysław Witek, Marek Bolanowski, Katarzyna Szamotulska, Agnieszka Wojciechowska-Luźniak, Aleksandra Jawiarczyk-Przybyłowska, Marcin Kałużny

**Affiliations:** ^1^ Department of Internal Medicine, Endocrinology and Diabetes, Mazovian Bródno Hospital, Medical University of Warsaw, Warsaw, Poland; ^2^ Department of Endocrinology, Diabetes and Isotope Therapy, Wroclaw Medical University, Wroclaw, Poland; ^3^ Department of Epidemiology and Biostatistics, Institute of Mother and Child, Warsaw, Poland

**Keywords:** acromegaly, diabetes, pasireotide long-acting release (LAR), pasireotide-associated hyperglycemia, glucose metabolism, metformin

## Abstract

**Objective:**

The aim of the study was to evaluate glucose metabolism, as measured by glycated hemoglobin (HbA1c) levels and the need for antidiabetic medical treatment, in patients with acromegaly resistant to first-generation somatostatin receptors ligands (SRLs) treated with pasireotide long-acting release (LAR) in real-world clinical practice. Biochemical control of acromegaly, as measured by growth hormone (GH) and insulin-like growth factor 1 (IGF-1) levels, was also assessed.

**Study Design:**

Two-center retrospective cohort of consecutive patients with acromegaly treated with first-generation SRLs at maximum doses, who had not achieved biochemical disease control. After SRLs were discontinued, patients were given pasireotide LAR 40 mg i.m. every 28 days. The dose was increased to 60 mg i.m. in patients for whom adequate control was not achieved after 3 months. Patients were given dietary and lifestyle advice, and antihyperglycemic treatment was modified as needed.

**Main Outcome Measures:**

Biochemical disease control parameters (GH and IGF-1 concentration), as well as HbA1c level at baseline and after 6 months.

**Results:**

In total, 39 patients with acromegaly were enrolled. GH concentration decreased (Δ_me_ =-1.56 µg/L, range -21.38–3.62, p <0.001) during 6 months of pasireotide LAR treatment. A worsening of metabolic status was observed, with an increase of median HbA1c (Δ_me_ =0.40%, range -0.20%–2.30%, p <0.001), and antihyperglycemic treatment intensification in 23 (59.0%) patients. The median decline in IGF-1 concentration was: -283.0 µg/L, range -682.7–171.6, p <0.001. IGF-1 reached the age- and gender-specific upper level of normal in 23 (59%) patients.

**Conclusions:**

Pasireotide LAR is an effective therapeutic option in patients with acromegaly refractory to first-generation SRLs. However, this therapy may result in pasireotide LAR-associated hyperglycemia, which requires early and aggressive antidiabetic medical therapy to prevent glucose homeostasis alterations.

## Introduction

Acromegaly is usually caused by pituitary adenoma secreting growth hormone (GH). Excess GH accompanied by increased insulin-like growth factor 1 (IGF-1) levels results in cardiovascular and metabolic complications. As the morbidity and mortality rate in acromegaly is significantly associated with IGF-1 and GH level, the aim of medical therapy is to ensure target IGF-1 level within age- and gender-specific normal ranges and to lower and normalize GH levels (1–4). Biochemical control of acromegaly assessed by a random GH level <2.5 µg/L in a recently published longitudinal study was associated with a significantly lower risk of developing diabetes mellitus as well as cardiovascular system disorders overall compared to those not controlled (5).

The treatment of choice is selective transsphenoidal adenomectomy performed by an experienced neurosurgeon. The average cure rate is 65%, but adenoma size, extrasellar extension, and cavernous sinus invasion are the most reliable factors predicting a complete tumor resection in individual patients (3, 4).

First-generation somatostatin receptor ligands (SRLs) (octreotide or lanreotide) are used either as preoperative or adjuvant therapy. In patients for whom this treatment failed (acromegaly resistant to first-generation SRLs), pasireotide long-acting release (LAR) is a reliable second-line therapeutic option. Pasireotide LAR is a multireceptor-targeted SRL, binding to four of five somatostatin receptor subtypes (SSTR 1,2,3,5), with the highest affinity for SSTR-5. Somatostatin receptors are also expressed in pancreatic islet cells; therefore, pasireotide LAR adversely affects glucose metabolism (6). This side effect of pasireotide LAR has been clearly identified in phase 3 clinical trials (2, 7) and discussed later by other authors (8, 9). However, the disease itself may destabilize glucose metabolism, as excess GH impairs insulin sensitivity and stimulates gluconeogenesis (10). Clinically, acromegaly is associated with cardiovascular and metabolic complications, and up to 50% of patients with acromegaly have prediabetes or diabetes (11, 12). Therefore, it is very important to understand the impact of pasireotide LAR on glucose metabolism not only in the clinical trial environment but also in daily clinical practice.

The aim of the study was to evaluate glucose metabolism, as measured by glycated hemoglobin A1c (HbA1c) levels and the need for antidiabetic medical treatment, in patients with acromegaly resistant to first-generation SRLs treated with pasireotide LAR in real-world clinical practice. Biochemical control of acromegaly, as measured by GH and IGF-1 levels, was also assessed.

## Materials and Methods

### Study Design and Participants

Between January 2019 and June 2020, we enrolled all 39 patients with acromegaly treated with first-generation SRLs at maximum doses, for whom biochemical disease control had not been achieved. Patients were from two tertiary referral centers. A history of type 1 diabetes mellitus was the only exclusion criterion from the study. During the recruitment of our patients switching to pasireotide LAR was the only option of second line medical therapy in Poland as the pegvisomant or cabergoline were not reimbursed by *the National Health Fund*. After discontinuation of treatment with SRLs, patients were given pasireotide LAR 40 mg i.m. every 28 days. The dose was increased to 60 mg i.m. in 24 patients (61.5%) for whom adequate control was not achieved after 3 months. Patients were given dietary and lifestyle advice, and antihyperglycemic treatment was modified when needed.

The study protocol was reviewed and approved by the local Bioethics Committees.

### Endpoints

HbA1c level and biochemical disease control parameters (GH and IGF-1 levels) were analyzed at baseline (V0) and after 6 months (V6). Using the diabetes diagnostic criteria published by the World Health Organization (13), patients were categorized into the following three groups based on the results of an oral glucose tolerance test (OGTT):

1. Normal glucose tolerance (NGT): fasting plasma glucose (FPG) ≤5.5 mmol/L and 120 min glucose <7.8 mmol/L
2. Prediabetes (IFG or IGT): FPG ≥5.6 mmol/L or 120 min glucose ≥7.8 mmol/L but <11.1 mmol/L
3. Diabetes: FPG ≥7.0 mmol/L or 120 min glucose ≥11.1 mmol/L.

OGTT was not performed at V0 if the diagnosis of diabetes was known earlier (prior to study entry; n=1). Additionally, at V6 OGTT was performed in all patients except for those with diabetes already diagnosed at V0 (n=4).

Additionally, patients were categorized by their acromegaly biochemical control status, defined as:

1. IGF-1 below upper level of normal (ULN) vs. IGF-1 ≥ULN
2. GH concentration below 2.5 µg/L vs. ≥2.5 µg/L (5, 7)
3. IGF-1 <ULN and GH concentration <2.5 µg/L vs. IGF-1 ≥ULN and GH concentration ≥2.5 µg/L.

### Laboratory Tests

HbA1c was measured using the ion-exchange high performance liquid chromatography (HPLC) method, certified by the National Glycohemoglobin Standardization Program (NGSP) and the International Federation of Clinical Chemistry (IFCC), and the Bio-Rad D-10 Hemoglobin Testing System^®^ (Bio-Rad Laboratories, CA, USA). The linear range was 18–179 mmol/mol (3.8%–18.5%) and coefficient of variation (CV) <2.5%.

Random serum GH was determined using the electrochemiluminescence (ECLIA) immunoassay on the Elecsys^®^ analyzer (Roche Diagnostics, Germany). GH was calibrated against the pure 22kDa recombinant growth hormone (WHO IRP 98/574). Functional sensitivity was 0.05 µg/L and limit of detection was 0.03 µg/L, respectively. Serum IGF-1 was measured by enzyme-linked immunosorbent assay (ELISA; LDN, Germany). Analytical sensitivity was 9.75 µg/L. IGF-1 values were compared with age- and gender-standardized normal values.

### Statistical Analysis

Descriptive statistics were used to present the study population at baseline. For categorical variables, the number and percentage of occurrences were reported; for continuous variables, the following were provided: mean and standard deviation (SD), median and the range as well as the number of observations.

The dynamics of evaluated parameters were analyzed using the Wilcoxon exact test and McNemar–Bowker test. Spearman’s Rho correlation coefficient (r_S_) and the Mann–Whitney exact test were applied to examine the relationships between changes in HbA1c and age, baseline GH or IGF-1 levels and gender.

All statistical analysis was performed using IBM^®^ SPSS^®^ Statistics 25. A p-value below 0.05 was considered statistically significant.

## Results

### Demographic and Clinical Characteristics at Baseline

In total, 39 patients with acromegaly were enrolled at two centers: 20 at one center, 19 at the other. Mean age was 47.2 ± 12.7 years, and 16 (41%) patients were female (**Table 1**).

**Table 1:** Baseline characteristics of the cohort.

	n = 39
Age, mean (SD), years	47.2 (12.7)
≥65 y/o, n (%)	5 (12.8)
Female, n (%)	16 (41.0)
Magnetic resonance imaging finding, n (%)	
Macroadenoma	39 (100.0)
Previous surgery, n (%)	
0	6 (15.4)
1	22 (56.4)
2	11 (28.2)
Previous irradiation^*^, n (%)	4 (10.3)
GH, median (range), µg/L	3.8 (0.6–27.3)
IGF-1, median (range)	
µg/L	491.0 (215.9–1198.5)
ULN	1.9 (1.03–4.86)
Glucose metabolism status, n (%)	
Normal glucose tolerance	15 (38.4)
Prediabetes	20 (51.3)
Diabetes	4 (10.3)
Antihyperglycemic treatment, n (%)	
Diet only	25 (64.1)
Metformin	14 (35.9)

GH, growth hormone; IGF-1, insulin-like growth factor–1; n, number of patients; SD, standard deviation; ULN, upper level of normal; y/o, years old.

^*^Irradiation: n = 1 in 2009, n = 1 in 2011, n = 2 in 2014.

All but six patients had a history of one or two transsphenoidal procedures. Median GH level was 3.8 µg/L (range 0.6–27.3) and in its concentration was below 2.5 µg/L in 14 patients (35.9%). Median IGF-1 was 491 µg/L (range 216–1199), and in all patients IGF-1 exceeded the ULN (range 1.0–4.9).

**Figure 1:**
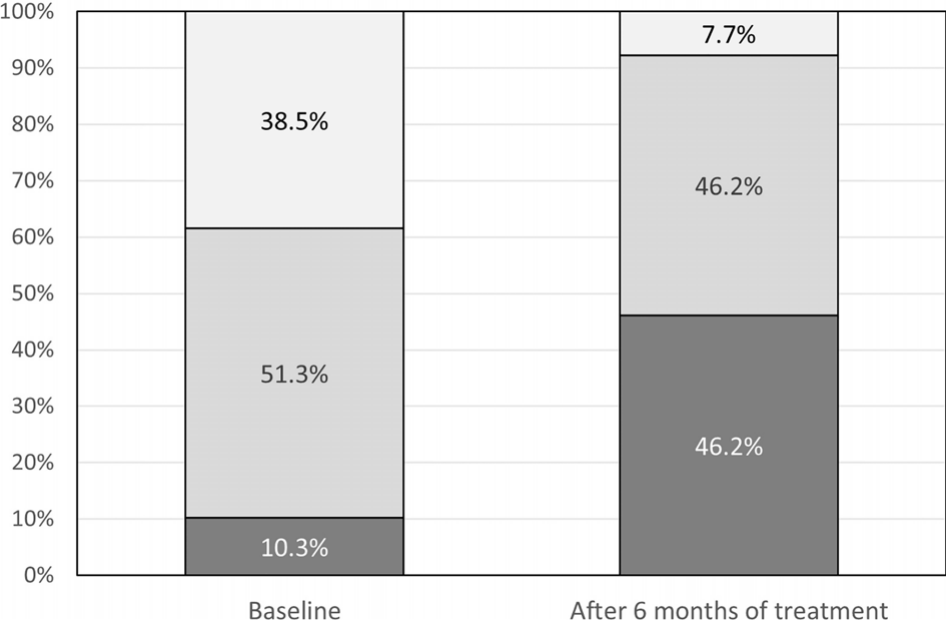
Glucose metabolism status at baseline and after 6 months of treatment: dark grey – diabetes, medium grey - prediabetes, light grey – normal glucose tolerance.

Normal glucose tolerance was observed in 15 (38.4%) patients. In total, 20 (51.3%) patients were diagnosed as prediabetic and were given dietary advice or metformin or both (dose range: 500–2,450 mg/d). Diabetes was present in four (10.3%) patients (**Figure 1**). Of these, two patients were treated with metformin only (1,000–1,500 mg/d), one was treated with metformin and insulin, and one with metformin and gliclazide. We observed a borderline association between increasing age and worsening glucose metabolism status (p_trend _=0.053). Median HbA1c percentage at baseline was 5.60% (range 5.0%–7.5%). HbA1c correlated with age (r_S_=0.44; p=0.005).

### Glucose Metabolism After 6 Months of Treatment

During the study period, the glucose metabolism status worsened (**Figure 1**, p<0.001): prediabetes progressed to diabetes in 12 of 20 (60%) patients; in 12 of 15 (80%) patients with normal glucose tolerance, prediabetes or diabetes had developed. Overall, three patients had normal glucose tolerance.

**Figure 2:**
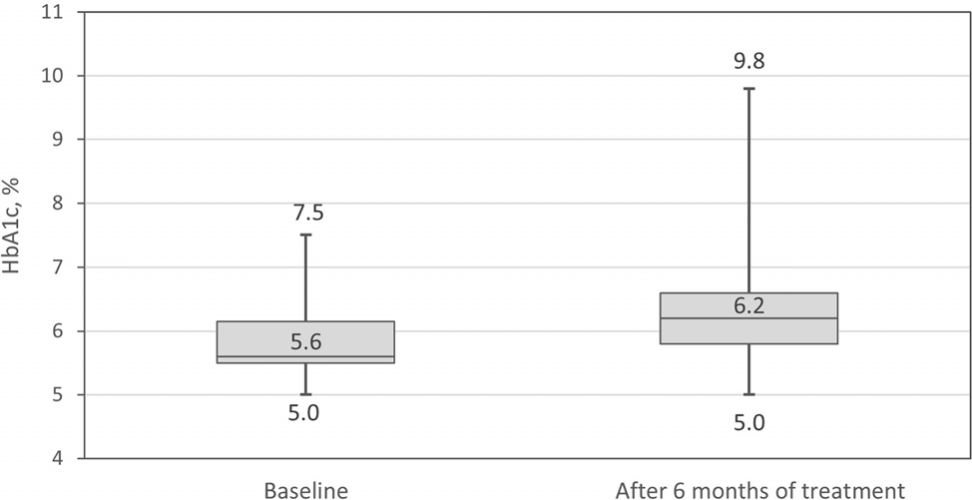
Glycated hemoglobin A1c (HbA1c) percentage at baseline and after 6 months of treatment.

After 6 months of treatment with pasireotide LAR median HbA1c increased by Δ_me_=0.40%, (range -0.20%–2.30%, p<0.001) (**Figure 2**); this change did not correlate with age (r_S_=0.056; p=0.736). The mean HbA1c percentage did not differ at 6 months between controlled and uncontrolled patients regardless of the criteria for control of acromegaly (IGF-1, GH, or a combination of both).

**Figure 3:**
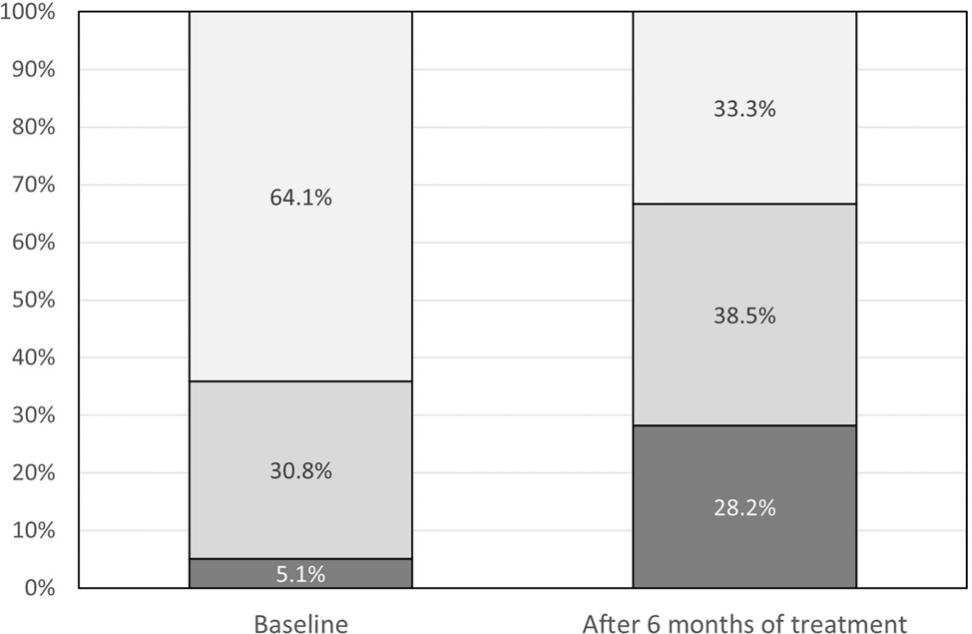
Antihyperglycemic treatment at baseline and after 6 months of treatment: dark grey – metformin and other drugs, medium gray – metformin only, light gray – diet only.

**Figure 4:**
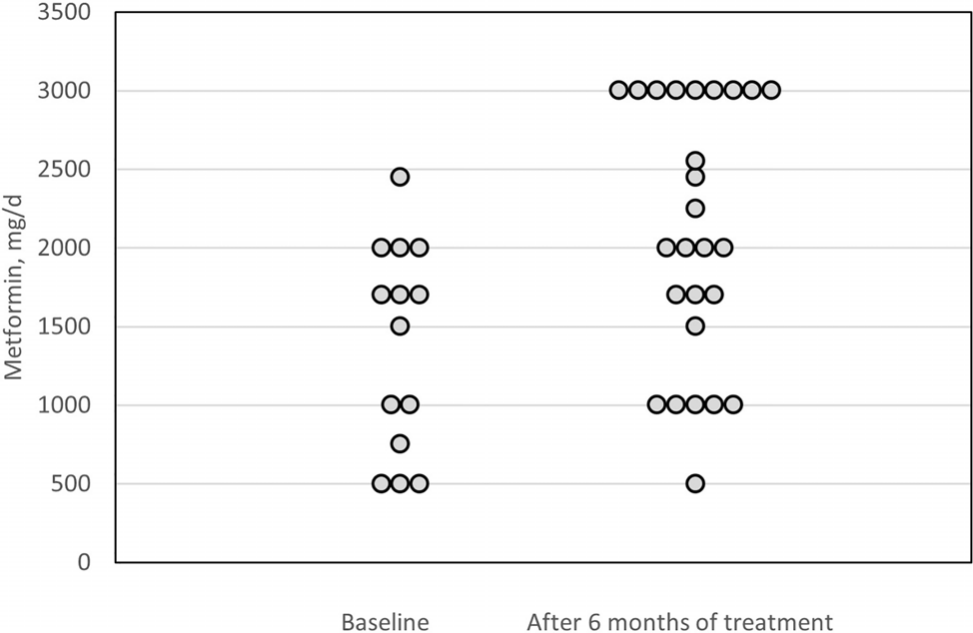
Metformin doses at baseline and after 6 months of treatment.

During 6 months of pasireotide LAR treatment, antihyperglycemic therapy was intensified in 23 (59%) patients (p<0.001) (**Figure 3**). In patients taking metformin at baseline, the median dose increased by 1,000 mg/day (range 0–2,000 mg/day; p<0.001). The number of patients taking metformin during the study increased from 14 to 26 (**Figure 4**), and in 10 patients additional drugs were introduced: glucagon-like peptide-1 (GLP-1) analogue, dipeptidyl peptidase 4 (DPP-4) inhibitor or basal insulin. Details of glucose metabolism status for individual patients who required more than one antidiabetic drug are presented in **Table 2**.

**Table 2:** Glucose metabolism status and management for 10 individual patients who required more than one antidiabetic drug after 6 months of treatment with pasireotide LAR.

Patient No.	Baseline			After 6 months			
	Diagnosis	HbA1c %	Antidiabetic treatment	Diagnosis	HbA1c %	Δ HbA1c %	Antidiabetic treatment
1	Prediabetes	6.2	Met 1700 mg/d	Diabetes	6.9	+0.7	Met 3000 mg/d + GLP-1 + Ins
2	Diabetes	6.8	Met 1000 mg/d	Diabetes	7.4	+0.6	Met 3000 mg/d + GLP-1 + Ins
3	Prediabetes	5.0	Met 2000 mg/d	Diabetes	6.1	+1.1	Met 3000 mg/d + Ins
4	Prediabetes	6.3	Met 1700 mg/d	Diabetes	6.4	+0.1	Met 3000 mg/d + Ins
5	Prediabetes	5.6	Diet	Diabetes	6.8	+1.2	Met 3000 mg/d + DPP-4
6	Prediabetes	5.6	Met 500 mg/d	Diabetes	6.3	+0.7	Met 2000 mg/d + Glic 60 mg/d
7	Prediabetes	6.3	Met 500 mg/d	Diabetes	7.8	+1.5	Met 2000 mg/d + Glic 30 mg/d
8	Prediabetes	6.4	Diet	Diabetes	6.6	+0.2	Met 2000 mg/d + Ins
9	Diabetes	7.5	Met 2000 mg/d + Glic 60 mg/d	Diabetes	9.8	+2.3	Met 3000 mg/d + Ins
10	Diabetes	6.3	Met 1500 mg/d	Diabetes	6.5	+0.2	Met 2000 mg/d + GLP-1

DPP-4, dipeptidyl peptidase 4 inhibitor; Glic, gliclazide; GLP-1, glucagon-like peptide-1 analog; HbA1c, glycated hemoglobin A1c; Ins, basal insulin; d, day; Met, metformin.

### Hormonal Assessment After 6 Months of Treatment

**Figure 5:**
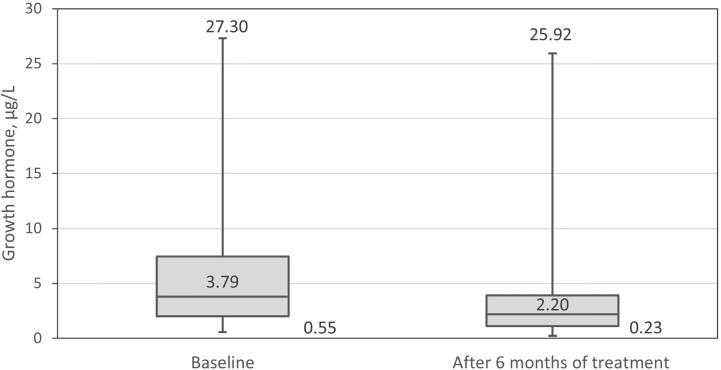
Growth hormone (GH) at baseline and after 6 months of treatment.

**Figure 6:**
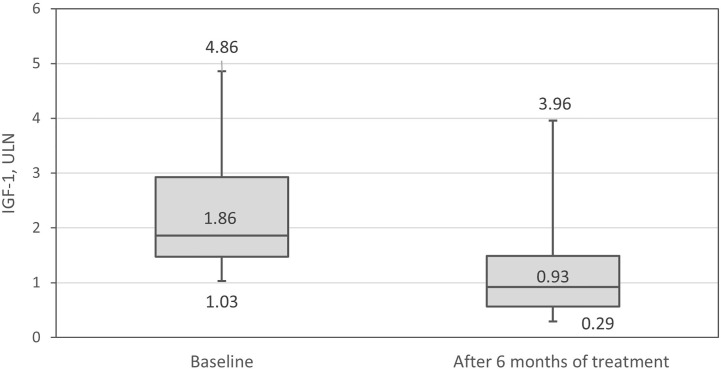
Insulin-like growth factor-1 (IGF-1) in relation to the upper level of normal (ULN) at baseline and after 6 months of therapy.

The GH level decreased significantly (Δ_me_ =-1.56 µg/L, range -21.4–3.62, p=0.001) during 6 months of pasireotide LAR treatment (**Figure 5**). The median decline in IGF-1 level was: -283.0 µg/L, range -682.7–171.6, p <0.001. IGF-1 expressed in ULN units decreased by median 1.05, range -3.06–0.72, p <0.001 (**Figure 6**). After adjustment for the centers, the differences remained statistically significant.

After 6 months of treatment 24 patients (61.5%) were receiving the 60 mg of pasireotide LAR, whereas remaining 15 patients (38.5%) the dose of 40 mg. The IGF-1 level within ULN and GH < 2.5 µg/L were confirmed in 23 (59.0%) and 21 (53.8%) patients, respectively, whereas both criteria were fulfilled in 16 (41%) patients (**Table 3**). If we adopted here a more stringent criterion for biochemical control of acromegaly based on GH level <1.0 µg/L (commonly used for postsurgical assessment) we would obtain a lower rate of GH normalization, namely in eight patients (20.5%). We observed no differences in the proportion of men and women who achieved biochemical control of acromegaly by the applied definitions. However, patients with biochemical control were older than those with no control (**Table 3**).

**Table 3:** Biochemical control of acromegaly—patient distribution and age.

	Controlled		Uncontrolled		P for difference in age
	N (%)	Mean (SD) age, years	N (%)	Mean (SD) age, years	
IGF-1 <ULN	23 (59.0)	50.6 (11.94)	16 (41.0)	42.2 (12.40)	0.040
GH <2.5 µg/L	21 (53.8)	51.1 (11.02)	18 (46.2)	42.5 (13.20)	0.032
IGF-1 <ULN and GH < 2.5 µg/L	16 (41.0)	53.1 (10.61)	23 (59.0)*	43.0 (12.53)	0.012

*n = 11 had both IGF-1 >ULN and GH >2.5 µg/L; n=7 had IGF-1 <ULN and GH ≥2.5 µg/L; n=5 had IGF-1 ≥ULN and GH < 2.5 µg/L.

GH, growth hormone; IGF-1, insulin-like growth factor-1; n, number of patients; SD, standard deviation; ULN, upper level of normal.

## Discussion

Pasireotide LAR is an effective treatment option for patients with acromegaly resistant to first-generation SRLs (7, 8). Our study in Polish clinical practice confirms these results. Improvement in acromegaly parameters (GH, IGF-1) was observed in all patients participating in the study. We found that patients who achieved disease biochemical control were on average older than those who did not, and this observation was consistent across the different definitions of disease control. A possible explanation could be the more aggressive course of the disease in younger patients than the older ones. A recently published retrospective analysis of 96 patients with acromegaly found similar differences; the authors suggested different acromegaly phenotypes: one in young patients who respond poorly to medical treatment and the other in older patients who respond well to medical therapy (14).

Our study confirms the increased risk of pasireotide LAR-associated impairment of glucose metabolism, which is well known and an expected adverse event of such a treatment, and has been observed in previous clinical trials (2, 7, 15). We found that after 6 months of treatment with pasireotide LAR, the proportion of patients with normal glucose metabolism decreased from almost 40% to less than 10%. These data should be viewed in light of two facts: one that diabetes is a very common complication in patients with acromegaly, and the other that medical treatment of acromegaly may result worsening of glucose metabolism. The prevalence of diabetes in this patient population is explained by the impact of GH on insulin sensitivity and gluconeogenesis (10) as already mentioned, and has been demonstrated by large epidemiological studies; e.g. Petrossians et al. showed that the prevalence of diabetes at the time of acromegaly diagnosis was 27.5% in the Liège Acromegaly Survey (16). In our cohort, this proportion was lower: slightly over 10% of patients were diagnosed with diabetes but more than 50% had prediabetes when the treatment with pasireotide LAR started. The lower proportion of patients with overt diabetes leads us to the second fact, namely effects of treatment. Even though our patients at the time of treatment switch were not controlled with first-generation SRLs, they were likely to have long-standing disease, successfully treated at least during certain periods. According to a meta-analysis of 31 studies including 619 patients, the first-generation SRLs had no effect on FPG or HbA1c (17), and pegvisomant may even moderately improve insulin sensitivity (18, 19).

As hyperglycemia and diabetes are long-term risk factors for cardiovascular disease, early and effective management of this complication is important. Furthermore, proper management of hyperglycemia increases the chance of maintaining long-term compliance with pasireotide LAR (20).

According to Samson (20), it is possible to predict which patients will develop diabetes based on FPG levels. In the Sheppard et al. study, diabetes (defined as glucose level >125 mg/dl) developed in 45% of patients with baseline FPG levels >100–125 mg/dl and in 19% of patients with baseline FPG levels <100 mg/dl (21). Similar findings were observed for baseline HbA1c level: 55% of patients with impaired glucose tolerance (HbA1c levels 5.7%–6.5%) developed diabetes, while in patients with normal glucose tolerance (HbA1c levels <5.7%) only 23% developed it (21). Other studies also show that baseline HbA1c may be the most important predictor of development of diabetes in patients treated with pasireotide LAR (22–25). Thus, baseline FPG and HbA1c levels should be assessed before starting and during pasireotide LAR therapy.

In the Colao et al. study, 44.4% of patients in the pasireotide LAR group received antidiabetic medications, with metformin and sulfonylureas the most commonly used (2). In our study, an antidiabetic drug had to be initiated in approximately one-third of patients. In addition, most patients who were treated with metformin at baseline required a dose increase and in several patients two or more antidiabetic drugs had to be administered.

The mechanism of pasireotide LAR-related hyperglycemia needs to be understood to introduce optimal treatment (20). Insulin-producing pancreatic β-cells mainly express SSTR-2 and SSTR-5; pasireotide LAR suppresses insulin secretion because of its high SSTR-5 affinity. Although glucagon-producing α-cells express SSTR-2, the pasireotide LAR inhibitory effect on glucagon secretion is only modest (10).

Considering the above, it is essential to closely monitor blood glucose levels in patients treated with pasireotide LAR. Hyperglycemia should be managed with proper antidiabetic drugs. No specific recommendation has been published for acromegaly patients with hyperglycemia (20). However, there are expert statements on short-acting pasireotide-induced hyperglycemia management in Cushing’s disease (2, 26). According to the summary of product characteristics for pasireotide LAR, the European Medicines Agency and Food and Drug Administration recommend managing pasireotide LAR-induced hyperglycemia with antidiabetic drugs, using standard guidelines for treatment of hyperglycemia (27, 28). Conforming to these guidelines, unless contraindicated, metformin is the preferred treatment for hyperglycemia (29). However, some studies show that DPP-4 inhibitors and GLP-1 receptor agonists are more effective options than metformin or sulfonylureas, and authors suggest them as the first-line treatment (20, 30). The maximum daily dose of metformin is 2550 mg in the USA and 3000 mg in the European Union. Additionally, doses above 2000 mg are generally associated with little additional efficacy and, in some cases, poorer tolerability (29). Therefore, it seems reasonable to consider adding an incretin-based therapy after reaching a dose of 2000 mg of metformin in order to increase the hypoglycaemic effect and avoid accumulation of metformin side effects. 

We have also found that the age of the patients positively correlated with HbA1c at baseline and that HbA1c was not affected by the acromegaly control status. This observation reflects the situation in the general population and is also consistent with the findings of the French Acromegaly Registry study (31).

We analyzed the impact of 6 months’ pasireotide LAR treatment on acromegaly parameters and glucose metabolism. Some studies show that FPG and HbA1c levels rise rapidly for the first 3 months and then remain stable without further antidiabetic therapy (7, 21, 23, 25). Further research is necessary, therefore, to confirm this in clinical practice.

Our study was conducted in clinical practice in Poland where, for years, only the first-generation SRLs (octreotide and lanreotide) were reimbursed for treatment of acromegaly (32, 33). GH-receptor antagonist pegvisomant, which is particularly useful in patients with associated diabetes was not financed from public funds at the time the study began. Therefore, when the pasireotide LAR treatment program started, patients generally presented with more advanced disease and with a relatively high prevalence of complications of acromegaly. In other words, their metabolic status and complications were greater than expected immediately after diagnosis of acromegaly resistant to first-generation SRLs. This is the probable explanation of why our cohort of patients was particularly prone to deterioration of their metabolic status. The other limitation of our study lies in its retrospective observational nature. This type of study allows generation of real-world evidence and informs the medical community about the effects of treatment in an unselected patient cohort; however, it does not allow for exclusion of bias and does not ensure robustness and completeness of data. For this reason, we decided to assess glucose metabolism with HbA1c and not FPG.

We confirm that treatment with pasireotide LAR may result in carbohydrate metabolism disorders, which in most patients requires intensification of antidiabetic therapy. Therefore, all patients treated with pasireotide LAR should be given dietary and lifestyle advice and have glucose-related parameters closely monitored. These findings do not undermine our other observation that pasireotide LAR is an effective therapeutic option in patients with acromegaly refractory to first-generation SRLs. Additionally, if properly managed, it is also a safe therapeutic option.

## Data Availability Statement

The raw data supporting the conclusions of this article will be made available by the authors, without undue reservation.

## Ethics Statement

The studies were conducted according to Good Clinical Practice guidelines and the Declaration of Helsinki. The studies involving human participants were reviewed and approved by Bioethics Committee at the Medical University of Warsaw, Warsaw, Poland. The patients/participants provided their written informed consent to participate in this study.

## Author Contributions

PW and MB conceived the concept of the study. PW, MK, AW-L, KS, AJ-P, and MB contributed to the study design. PW, MK, and KS organized the database. KS performed the statistical analysis. PW, MK, KS, and MB wrote the manuscript. All authors contributed to the article and approved the submitted version.

## Funding

The National Health Fund covered the costs of treatment as well as laboratory and imaging tests for all study patients. The authors declare that this study received funding from *Recordati Rare Diseases*. The funder provided medical editorial assistance and provided article processing fee. The funder had no role in the study design, data collection and analysis, preparation of the manuscript, or decision to publish.

## Conflict of Interest

The authors declare that the research was conducted in the absence of any commercial or financial relationships that could be construed as a potential conflict of interest.
